# The global burden of traumatic brain injury in adolescents and young adults, 1990–2021: a systematic analysis for the Global Burden of Disease Study 2021

**DOI:** 10.1080/07853890.2026.2656513

**Published:** 2026-04-10

**Authors:** Yunxiang Chen, Jie Yang, Na Zhang, Mingfeng Tong, Lin Chen, Ya Li, Wentao Dong, Xinyu Li, Yongye Wang, Binbin Ren, Kai Zhang

**Affiliations:** ^a^ Department of Neurosurgery, Affiliated Jinhua Hospital, Zhejiang University School of Medicine, Jinhua, China; ^b^ Department of Emergency Medicine, Sir Run Run Shaw Hospital, Zhejiang University School of Medicine, Hangzhou, China; ^c^ Department of Dermatology, Affiliated Jinhua Hospital, Zhejiang University School of Medicine, Jinhua, China; ^d^ College of Mathematical Medicine, Zhejiang Normal University, Jinhua, China; ^e^ Department of Orthopedics, Yongkang Sixth People’s Hospital (Yongkang Orthopedic Hospital), Yongkang, China; ^f^ Department of Traditional Chinese Medicine, The People’s Hospital of Cangnan Zhejiang, Wenzhou Medical University, Wenzhou, China; ^g^ Department of Infectious Diseases, Affiliated Jinhua Hospital, Zhejiang University School of Medicine, Jinhua, China; ^h^ Department of Critical Care Medicine, Second Affiliated Hospital, Zhejiang University School of Medicine, Hangzhou, China

**Keywords:** Traumatic brain injury, global burden, adolescents, young adults, socio-demographic index, prevalence, years lived with disability

## Abstract

**Background:**

Traumatic brain injury (TBI) remains a leading cause of global disability among young adults, with burdens distributed unevenly across socio-demographic contexts.

**Methods:**

Using Global Burden of Disease 2021 data, we analyzed TBI prevalence and years lived with disability (YLDs) for individuals aged 15–49 years across 204 countries (1990–2021). Age-standardized rates were calculated via Bayesian meta-regression (DisMod-MR 2.1), and temporal trends were assessed using the estimated annual percentage change (EAPC). Associations with the Socio-demographic Index (SDI) were examined via correlation and regression.

**Results:**

Globally, prevalent TBI cases rose from 12.53 million in 1990 to 14.99 million in 2021. Conversely, age-standardized prevalence and YLD rates declined (EAPC –0.81 and –0.78, respectively). Geographically, rates increased in high-income Asia Pacific and North America but decreased elsewhere. Prevalence was consistently higher in males, peaking in the 45–49 age group. Road injuries and falls were the primary risk factors; these dominated in high-SDI regions, whereas conflict-related injuries prevailed in low-SDI settings. Age-standardized rates correlated positively with SDI (*p*<0.001).

**Conclusion:**

Although age-standardized TBI rates have decreased, the absolute burden has grown due to population growth and shifting demographics. These findings emphasize the necessity of age-, sex-, and region-specific prevention and rehabilitation strategies to mitigate the ongoing impact of TBI.

## Introduction

Traumatic brain injury (TBI) is a major global health concern and a leading cause of long-term disability across the life course [1,2]. Adolescence and young adulthood are key periods of exposure to injury-related risks, with TBI commonly arising from road traffic collisions, interpersonal violence, and occupational hazards; falls also contribute materially within this age range—particularly during adolescence—although the highest fall burden overall occurs in very young children and older adults [3,4]. At this stage of life, TBI can disrupt education, employment, and social functioning, and may lead to persistent physical, cognitive, and psychosocial sequelae [5,6]. Clinically, TBI is often classified into mild and moderate-to-severe forms with different prognoses and care needs [7,8], yet the global non-fatal burden of these severity strata among adolescents and young adults remains incompletely characterised [9].

Despite increasing recognition of TBI as a public health priority, global evidence describing age- and sex-specific patterns, severity profiles, and sociodemographic disparities remains fragmented—particularly for adolescents and young adults [1,10]. Many existing estimates are restricted to single-country analyses or hospital-based cohorts, limiting generalisability and obscuring cross-setting differences in injury prevention, trauma care capacity, and access to rehabilitation [11–13].

To address these gaps, we used Global Burden of Disease (GBD) 2021 estimates to quantify TBI prevalence and years lived with disability (YLDs) from 1990 to 2021 across 204 countries and territories, stratified by age, sex, and TBI severity [14]. We further assessed temporal trends and disparities by Socio-demographic Index (SDI), providing policy-relevant evidence to inform targeted prevention, surveillance, and rehabilitation strategies for adolescents and young adults.

## Methods

### Data sources and study framework

This study is a systematic analysis leveraging the GBD Study 2021 framework, which provides comprehensive global, regional, and national estimates of the burden of TBI from 1990 to 2021. It is a repeated cross-sectional secondary analysis of GBD 2021 estimates and was not prospectively registered as a PRISMA-type systematic review (e.g. in PROSPERO). This design evaluates population-level burden across successive calendar years (1990–2021) rather than following individuals longitudinally. The GBD study employs a standardised comparative risk assessment methodology to ensure the comparability of estimates across locations, time periods, and socio-demographic contexts [14,15].

The GBD data acquisition strategy integrates data from systematic reviews, hospital and registry records, population-based surveys, and national health databases [16]. Public access to these estimates is available through the Global Health Data Exchange (GHDx) Results Tool. For this study, data were extracted from the GBD 2021 Results Tool hosted on GHDx (http://ghdx.healthdata.org/gbd-results-tool) using the following parameters: cause, traumatic brain injury; measures, prevalence and YLDs; metrics, number and rate; age groups, 15–19, 20–24, 25–29, 30–34, 35–39, 40–44, and 45–49 years; sex, male, female, and both; years, 1990–2021; and all available locations, including 204 countries and territories, GBD regions, and super-regions. Extracted outputs included age-specific counts, age-standardised rates, and corresponding 95% uncertainty intervals.

TBI burden estimates were derived from mortality and morbidity data using a cause-of-injury framework, with cases identified based on International Classification of Diseases (ICD-9 and ICD-10) codes [14,15]. This study did not generate de novo epidemiological estimates, but analysed published GBD 2021 summary outputs produced within the standard GBD modelling framework. The study population comprised individuals aged 15–49 years, stratified into seven 5-year age groups. The SDI, a composite indicator of educational attainment, total fertility rate, and income per capita, was used to classify countries and territories into five SDI quintiles (low, low-middle, middle, high-middle, and high). We applied the standard IHME/GBD SDI quintile cut-points to ensure comparability across locations and over time [15,17].

### Classification of TBI severity

The classification of TBI severity in this study follows internationally recognized clinical and epidemiological criteria, including World Health Organization (WHO) guidelines [18], Glasgow Coma Scale thresholds [19], and methodologies from previous GBD studies [4]. To ensure consistency with the GBD 2021 injury modelling framework and comparability across locations and time, we adopted a pragmatic epidemiological stratification (minor vs moderate-to-severe) based largely on available severity information, rather than a comprehensive multidimensional clinical taxonomy.

Total TBI burden encompasses all severities, integrating both minor and moderate-to-severe cases to provide a comprehensive estimate of TBI at global, regional, and national levels.Minor TBI, typically classified as mild TBI, corresponds to a GCS score of 13–15, often reflecting a milder acute clinical presentation; however, ‘mild’ TBI can still require hospitalisation, may present with neuroimaging abnormalities, and can be associated with persistent post-injury symptoms. Minor TBI can still result in short- and longer-term cognitive or neuropsychological impairment and increased healthcare utilisation.Moderate-to-severe TBI, defined by a GCS score of ≤12, includes intracranial injuries, skull fractures, and severe neurotrauma, frequently leading to long-term disability, functional impairment, or mortality, necessitating intensive medical care and rehabilitation, while recognising that individual recovery trajectories can vary and are not fully captured by categorical severity labels.

We acknowledge emerging moves beyond the mild–moderate–severe paradigm toward multidimensional TBI characterisation (clinical features, imaging, biomarkers, and contextual modifiers). However, such harmonised patient-level inputs are not consistently available in the data informing GBD estimates; thus, our severity categories should be interpreted as a GBD-compatible construct for comparability rather than a definitive clinical taxonomy, and future work would benefit from improved data harmonisation.

### Statistical analysis

In GBD 2021, TBI burden estimates were generated using DisMod-MR 2.1, a Bayesian meta-regression tool used in the GBD study to synthesize epidemiological data while adjusting for data sparsity, underreporting, and measurement bias [15]. The prevalence and YLD estimates analysed in this study were obtained directly from published GBD 2021 modelled estimates rather than being independently re-estimated. Because our analyses were based on published, modelled GBD 2021 summary estimates (rather than individual-level survey microdata), conventional sampling weights are not applicable at this stage. Instead, the estimates should be interpreted as modelled population-level estimates produced within the GBD framework for each location, age group, sex, and year.

Age-standardised rates (ASR) for prevalence and YLDs were calculated by weighting age-specific rates using the GBD standard population, to account for differences in age structure across regions. These standard population weights were used to derive ASPR and age-standardised YLD rates for comparability across locations and over time; no additional study-level weighting is required when analysing GBD modelled outputs. YLD estimates were taken directly from GBD 2021, in which YLDs are computed using GBD disability weights. The formula used for ASR computation was:

$$\text{Age}-\text{standardised rate}=\frac{\sum_{i=1}^{N}a_{i}w_{i}}{\sum_{i=1}^{N}w_{i}}$$

where $a_{i}$ is the age-specific rate in the i-th age group, and $w_{i}$ represents the number of people in the same age group among the GBD standard population, and N is the number of age groups.

Trends in prevalence and YLDs rate from 1990 to 2021 were analyzed using Joinpoint regression models, which identify statistically significant inflection points in temporal trends [15]. The estimated annual percentage change (EAPC) was calculated using log-linear regression:

$$\text{EAPC}=100\times (e^{\beta }-1)$$

where β is the slope of the natural logarithm of the ASR over time. A positive EAPC indicates an increasing trend, while a negative EAPC indicates a decline.

The relationship between TBI burden and SDI levels was assessed using Spearman’s rank correlation coefficient, and locally weighted scatterplot smoothing regression was applied to visualize deviations from expected burden based on SDI levels. SDI quintiles were defined using the standard GBD reference cut-points provided by IHME/GBD [17].

All estimates were reported with 95% uncertainty intervals (UI), derived from 1,000 Monte Carlo simulations, accounting for variability in input data, model assumptions, and parameter estimates. In this study, ‘prevalent cases’ refer to the estimated number of individuals living with TBI-related health states in a given calendar year (i.e. prevalent TBI-related disability during that year), as defined within the GBD framework.

All data analyses were conducted in R (version 4.0.2) Visualizations of trends, geographic variations, and SDI-related disparities were generated using the ggplot2 package in R. Statistical significance was set at *p* < 0.05.

### Ethical considerations

This study is a secondary analysis of fully anonymized and publicly available data from the Global Burden of Disease Study 2021. As such, the Institutional Review Board of Affiliated Jinhua Hospital, Zhejiang University School of Medicine issued a formal waiver for ethical approval for this work. The research was conducted in accordance with the principles of the Declaration of Helsinki.

## Results

### Global burden of TBI, 1990–2021

Globally, prevalent TBI cases increased from 12,533,276 in 1990 to 14,992,354 in 2021 (Table 1). Over the same period, the ASPR declined from 462.40 to 379.69 per 100,000, corresponding to an EAPC of −0.81. YLDs attributable to TBI rose from 1,869,428 in 1990 to 2,248,036 in 2021 (Table 2), while the age-standardised YLD rate decreased from 68.97 to 56.93 per 100,000 (EAPC −0.78).

**Table 1.** Prevalent cases and ASPR of TBI in 1990 and 2021, and EAPC in ASPR, by global, SDI, and regional level.

Characteristic	1990		2021		EAPC, 1990–2021 (95%CI)
	Prevalence cases in 1990 (95%UI)	ASPR per 100,000 in 1990 (95%UI)	Prevalence cases in 2021 (95%UI)	ASPR per 100,000 in 2021 (95%UI)	
**Global**	1,253,3276 (11,970,186, 13,183,050)	462.40 (441.63, 486.37)	14,992,354 (14,244,721, 15,818,960)	379.69 (360.76, 400.62)	−0.81 (−0.88, −0.73)
**SDI level**					
Low SDI	648,953 (583,133, 761,941)	293.59 (263.81, 344.70)	1,451,471 (1,310,319, 1,654,309)	267.61 (241.59, 305.01)	−0.38 (−0.49, −0.27)
Low-middle SDI	1,891,744 (1,795,008, 2,017,401)	343.27 (325.72, 366.07)	3,126,116 (2,973,127, 3,297,442)	307.61 (292.56, 324.47)	−0.44 (−0.50, −0.37)
Middle SDI	4,038,241 (3,849,516, 4,253,834)	443.47 (422.74, 467.15)	5,091,303 (4,833,590, 5,382,139)	405.66 (385.13, 428.83)	−0.43 (−0.50, −0.36)
High-middle SDI	3,569,826 (3,405,884, 3,738,984)	632.49 (603.44, 662.46)	3,309,845 (3,150,054, 3,480,815)	525.73 (500.35, 552.88)	−0.90 (−1.03, −0.76)
High SDI	2,368,714 (2,248,297, 2,503,728)	513.98 (487.86, 543.28)	1,998,234 (1,902,580, 2,109,770)	397.87 (378.82, 420.08)	−1.01 (−1.06, −0.96)
**Regions**					
Andean Latin America	70,035 (65,459, 75,943)	375.82 (351.26, 407.52)	124,617 (118,594, 130,726)	356.27 (339.05, 373.74)	−1.80 (−1.94, −1.65)
Australasia	69,100 (64,217, 74,951)	640.35 (595.09, 694.57)	69,478 (63,996, 76,030)	481.14 (443.18, 526.51)	−1.68 (−1.79, −1.56)
Caribbean	69,314 (66,171, 72,741)	379.46 (362.26, 398.22)	114,376 (102,628, 131,203)	477.66 (428.60, 547.94)	−1.31 (−1.41, −1.20)
Central Asia	195,625 (186,023, 205,184)	586.64 (557.85, 615.31)	239,279 (227,277, 251,673)	490.74 (466.13, 516.16)	−1.26 (−1.35, −1.16)
Central Europe	624,749 (593,279, 660,307)	1006.09 (955.41, 1063.36)	410,813 (389,419, 436,954)	779.68 (739.08, 829.29)	−1.14 (−1.37, −0.92)
Central Latin America	546,706 (515,540, 584,140)	669.75 (631.57, 715.61)	705,023 (673,276, 738,701)	529.59 (505.74, 554.88)	−1.02 (−1.22, −0.82)
Central Sub-Saharan Africa	69,633 (64,530, 768,80)	285.19 (264.29, 314.87)	177,633 (161,567, 198,611)	272.44 (247.80, 304.61)	−0.98 (−1.05, −0.90)
East Asia	2,722,362 (2,598,217, 2,870,050)	395.20 (377.18, 416.64)	2,919,535 (2,762,644, 3,083,725)	424.04 (401.25, 447.89)	−0.98 (−1.03, −0.93)
Eastern Europe	1,408,783 (1,333,232, 1,488,227)	1277.37 (1208.87, 1349.41)	991,321 (936,531, 1,050,774)	1030.20 (973.26, 1091.99)	−0.83 (−0.89, −0.76)
Eastern Sub-Saharan Africa	236,257 (200,880, 292,547)	283.24 (240.83, 350.72)	442,190 (395,580, 512,791)	211.17 (188.91, 244.89)	−0.79 (−0.86, −0.71)
High-income Asia Pacific	413,653 (390,435, 441,179)	445.63 (420.62, 475.29)	223,020 (208,466, 238,889)	285.12 (266.51, 305.41)	0.79 (0.66, 0.93)
High-income North America	712,524 (671,542, 761,780)	478.12 (450.62, 511.17)	505,523 (478,475, 538,363)	299.72 (283.68, 319.19)	0.73 (0.69, 0.76)
North Africa and Middle East	1,019,564 (928,662, 1,152,040)	636.14 (579.43, 718.80)	1,920,447 (1,773,046, 2,103,146)	574.41 (530.33, 629.06)	−0.33 (−0.51, −0.14)
Oceania	9231 (8766, 9763)	288.89 (274.34, 305.54)	25,390 (23,893, 27,146)	358.87 (337.71, 383.69)	−0.32 (−0.37, −0.27)
South Asia	1,519,491 (1,441,586, 1,607,456)	287.22 (272.49, 303.85)	2,776,604 (2,620,379, 2,943,805)	275.81 (260.29, 292.42)	−0.32 (−0.36, −0.29)
Southeast Asia	929,217 (866,389, 1,048,226)	392.74 (366.19, 443.04)	1,170,763 (1,098,817, 1,247,372)	315.74 (296.33, 336.40)	−0.31 (−0.54, −0.07)
Southern Latin America	97,566 (90,659, 104,546)	398.38 (370.18, 426.88)	138,960 (129,451, 149994)	400.61 (373.19, 432.42)	−0.26 (−0.36, −0.16)
Southern Sub-Saharan Africa	159,742 (148,525, 172,324)	620.19 (576.64, 669.04)	195,983 (182,968, 210,547)	453.97 (423.83, 487.71)	−0.25 (−0.33, −0.17)
Tropical Latin America	543,042 (511,144, 573,493)	691.60 (650.98, 730.38)	759,620 (717,005, 804,835)	633.92 (598.35, 671.65)	−0.18 (−0.30, −0.06)
Western Europe	942,313 (887,645, 1,008,604)	487.19 (458.93, 521.47)	640,710 (598,333, 690,320)	339.88 (317.40, 366.20)	−0.08 (−0.25, 0.09)
Western Sub-Saharan Africa	174,368 (166,273, 183,739)	203.69 (194.23, 214.63)	441,069 (416,749, 470,793)	192.34 (181.74, 205.30)	0.02 (−0.10, 0.13)

TBI: Traumatic brain injury; ASPR: age-standardised prevalence rate; EAPC: estimated annual percentage change; SDI: sociodemographic index.

**Table 2.** YLDs cases and age-standardised YLDs rate of TBI in 1990 and 2021, and EAPC in age-standardised YLDs rate, by global, SDI, and regional level.

Characteristic	1990		2021		EAPC, 1990–2021 (95%CI)
	YLDs cases, 1990 (95%UI)	Age-standardised rate per 100,000 population, 1990 (95%UI)	YLDs cases, 2021 (95%UI)	Age-standardised rate per 100,000 population, 2021 (95%UI)	
**Global**	1,869,428 (1,305,115, 2,532,518)	68.97 (48.15, 93.43)	2,248,036 (1,575,901, 3,024,070)	56.93 (39.91, 76.59)	−0.78 (−0.85, −0.71)
**SDI regions**					
Low SDI	97,358 (69,743, 128,310)	44.05 (31.55, 58.05)	218,438 (158,288, 285,ge-standardised rate per 100, adings as set inf are accurate.249)	40.27 (29.18, 52.59)	−0.36 (−0.47, −0.25)
Low-middle SDI	285,413 (200,977, 384,617)	51.79 (36.47, 69.79)	472,906 (333,627, 635,067)	46.53 (32.83, 62.49)	−0.42 (−0.49, −0.35)
Middle SDI	616,014 (429,224, 831,602)	67.65 (47.14, 91.32)	773,276 (544,639, 1,039,915)	61.61 (43.40, 82.86)	−0.43 (−0.50, −0.37)
High-middle SDI	536,695 (373,966, 740,917)	95.09 (66.26, 131.27)	497,504 (343,211, 681,540)	79.02 (54.51, 108.25)	−0.89 (−1.02, −0.75)
High SDI	331,572 (231,365, 451,038)	71.95 (50.20, 97.87)	283,597 (198,818, 388,472)	56.47 (39.59, 77.35)	−0.96 (−1.01, −0.90)
**Regions**					
Andean Latin America	97,263 (66,744, 131,767)	65.27 (44.79, 88.42)	68,637 (48,048, 92,730)	40.69 (28.49, 54.98)	−1.81 (−1.94, −1.67)
Australasia	56,544 (39,530, 77,331)	60.91 (42.59, 83.31)	30,402 (21,205, 41,576)	38.87 (27.11, 53.15)	−1.69 (−1.80, −1.57)
Caribbean	128,359 (89,537, 174,529)	66.36 (46.29, 90.23)	86,985 (59,927, 120,663)	46.14 (31.79, 64.01)	−1.32 (−1.43, −1.21)
Central Asia	24,389 (16,652, 33,239)	94.69 (64.65, 129.05)	29,660 (20,341, 40,504)	68.71 (47.12, 93.82)	−1.28 (−1.37, −1.19)
Central Europe	212,891 (148,683, 294,215)	193.03 (134.81, 266.77)	149,390 (104,376, 205,514)	155.25 (108.47, 213.57)	−1.13 (−1.35, −0.92)
Central Latin America	35,638 (25,775, 47,066)	42.73 (30.90, 56.43)	66,501 (47,835, 86,946)	31.76 (22.84, 41.52)	−1.01 (−1.20, −0.82)
Central Sub-Saharan Africa	9244 (6474, 12,427)	85.66 (60.00, 115.16)	9272 (6462, 12,543)	64.21 (44.75, 86.86)	−0.99 (−1.06, −0.91)
East Asia	94,078 (65,126, 129,851)	151.50 (104.88, 209.11)	61,772 (4,2504, 85,031)	117.24 (80.67, 161.38)	−0.98 (−1.03, −0.93)
Eastern Europe	141933 (99688, 189611)	59.99 (42.13, 80.14)	178,514 (126,674, 236,578)	48.14 (34.16, 63.80)	−0.83 (−0.90, −0.76)
Eastern Sub-Saharan Africa	29739 (20596, 40828)	89.18 (61.76, 122.44)	36,216 (25,230, 49,493)	74.28 (51.75, 101.51)	−0.80 (−0.87, −0.73)
High-income Asia Pacific	10,535 (7229, 14,409)	57.67 (39.57, 78.88)	17,223 (12,487, 22,267)	71.93 (52.15, 92.99)	0.77 (0.62, 0.92)
High-income North America	1411 (975, 1938)	44.17 (30.52, 60.66)	3883 (2795, 5323)	54.88 (39.51, 75.24)	0.72 (0.68, 0.77)
North Africa and Middle East	82,763 (58,492, 111,724)	101.39 (71.66, 136.87)	106,815 (74,265, 145,138)	80.24 (55.79, 109.02)	−0.33 (−0.51, −0.15)
Oceania	154,391 (111,710, 200,161)	96.33 (69.70, 124.89)	290,156 (207,505, 381,799)	86.79 (62.07, 114.20)	−0.31 (−0.37, −0.26)
South Asia	81,977 (57,215, 112,609)	104.40 (72.87, 143.42)	114,707 (79,821, 155,230)	95.72 (66.61, 129.54)	−0.31 (−0.34, −0.27)
Southeast Asia	10,437 (7445, 13,552)	42.75 (30.49, 55.50)	26,702 (19,108, 34,901)	40.95 (29.31, 53.53)	−0.30 (−0.53, −0.07)
Southern Latin America	228,207 (159,831, 313,708)	43.14 (30.21, 59.30)	418,252 (296,142, 564,246)	41.55 (29.42, 56.05)	−0.25 (−0.35, −0.15)
Southern Sub-Saharan Africa	26,336 (18,129, 35,948)	30.76 (21.18, 41.99)	66,948 (46,717, 90,032)	29.20 (20.37, 39.26)	−0.22 (−0.30, −0.14)
Tropical Latin America	10,645 (7579, 14,106)	57.12 (40.67, 75.69)	18,890 (13,134, 26,034)	54.00 (37.55, 74.43)	−0.18 (−0.30, −0.06)
Western Europe	419,496 (292,285, 575,501)	60.90 (42.43, 83.54)	448,411 (309,784, 614,072)	65.13 (44.99, 89.19)	−0.08 (−0.25, 0.08)
Western Sub-Saharan Africa	13,152 (9134, 17,779)	53.70 (37.30, 72.60)	18,699 (13,218, 25,237)	53.91 (38.11, 72.76)	0.02 (−0.09, 0.13)

TBI: Traumatic brain injury; YLDs: years lived with disability; EAPC: estimated annual percentage change; SDI: sociodemographic index.

### Regional and national burden of TBI, 1990–2021

Marked geographical heterogeneity was observed in both ASPR and age-standardised YLD rates (Tables 1 and 2). While most regions experienced declining ASPR and age-standardised YLD rates, High-income Asia Pacific and High-income North America showed increasing ASPR (EAPC 0.79 and 0.73, respectively) and parallel increases in age-standardised YLD rates (EAPC 0.77 and 0.72). The steepest declines in both ASPR and YLD rates were seen in Andean Latin America, Australasia, and the Caribbean.

**Figure 1.**
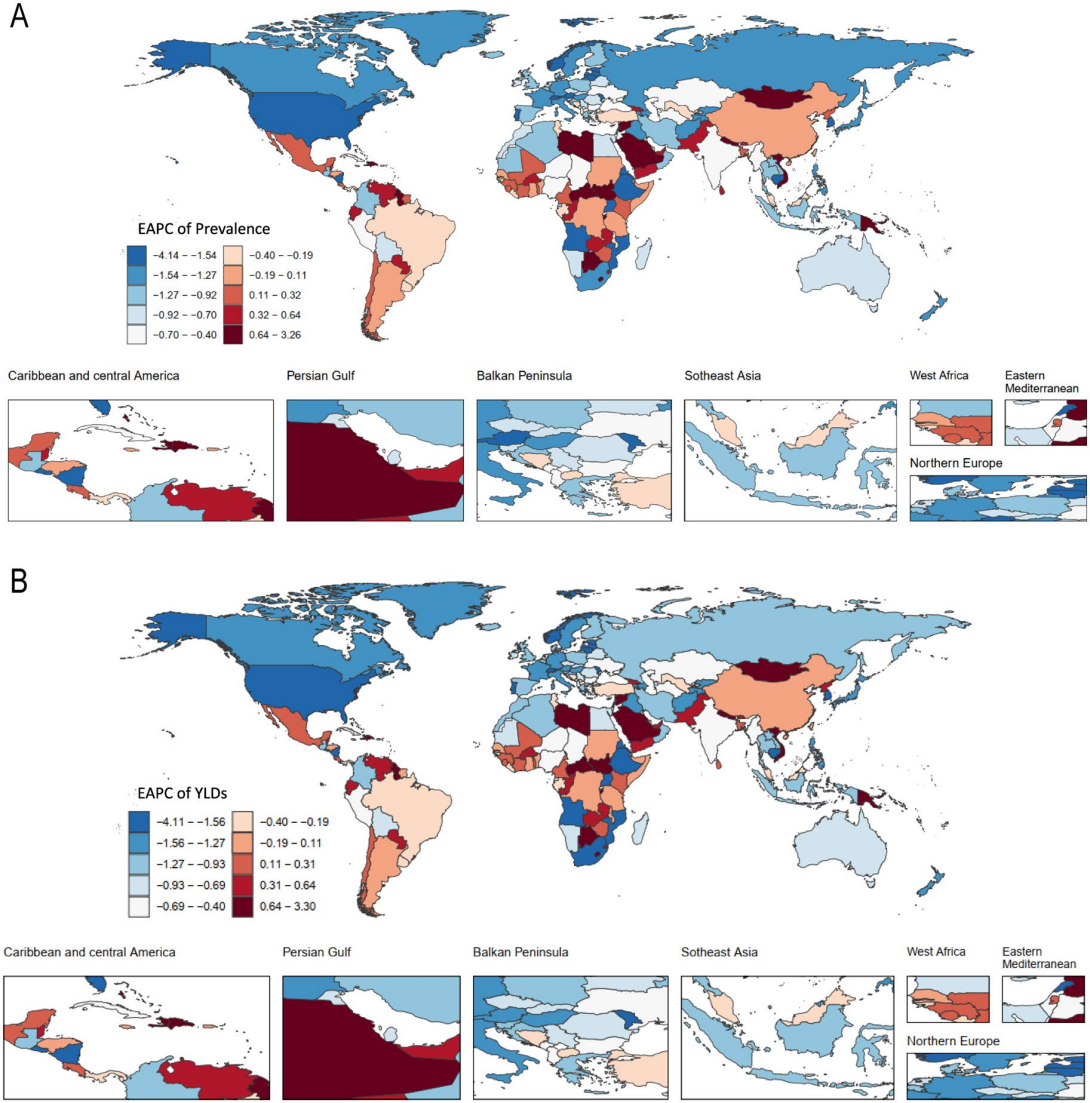
The EAPC of ASPR (A) and age-standardised YLDs rate (B) for TBI by national in 2021. TBI: Traumatic brain injury; ASPR: age-standardised prevalence rate; YLDs: years lived with disability; EAPC: estimated annual percentage change.

Distinct national variations in TBI prevalence and YLDs were observed (Figure 1). In the Syrian Arab Republic, prevalent TBI cases increased from 20,563 (95% UI 18,545–25,442) in 1990 to 57,558 (39,478–83,730) in 2021, accompanied by a marked rise in ASPR (EAPC 3.23; 95% CI 2.34–4.13). Over the same period, YLDs increased from 3,117 (2199–4282) to 8464 (6170–11,741), with a corresponding increase in the age-standardised YLD rate (EAPC 3.27; 2.36–4.18). Burundi also showed substantial increases (ASPR EAPC 3.17; 1.67–4.68; age-standardised YLD rate EAPC 2.92; 1.42–4.45). By contrast, pronounced declines were observed in Eritrea (ASPR EAPC −4.09; age-standardised YLD rate EAPC −4.06), followed by Lebanon (ASPR −2.87; YLD rate −2.85) and Nicaragua (ASPR −2.83; YLD rate −2.80).

### Age- and sex-specific patterns of TBI, minor TBI moderate-to-severe TBI burden in 2021

**Figure 2.**
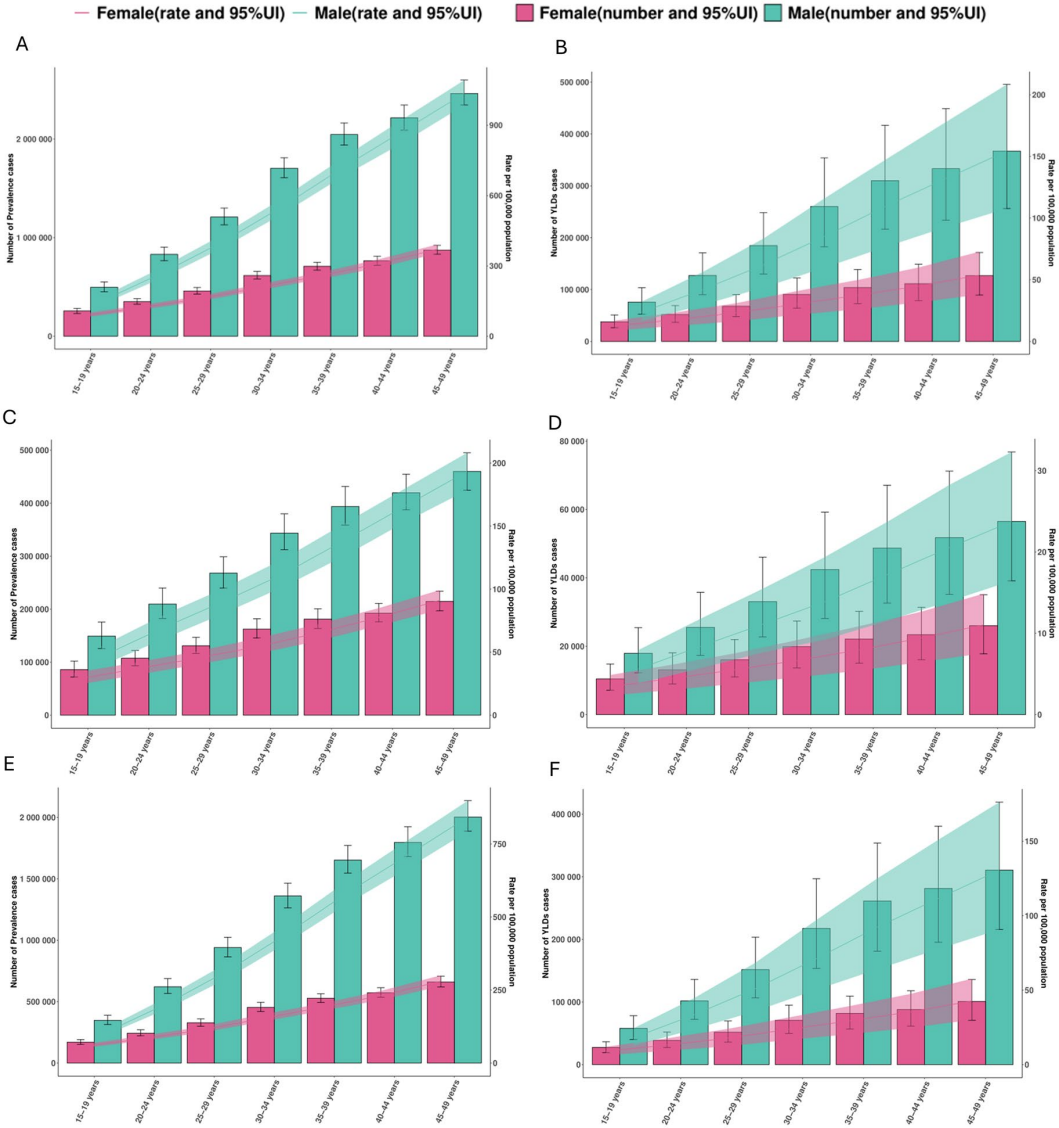
Global trends in the number of prevalence cases, ASPR, number of YLDs, and age-standardised YLDs rate by age and sex for TBI, minor TBI, and moderate-to-severe TBI. Panels A and B show the ASPR and age-standardised YLDs rate for total TBI, respectively. Panels C and D present corresponding data for minor TBI, and panels E and F for moderate-to-severe TBI. ASPR and age-standardised YLDs rate for females and males are illustrated with red and green lines, respectively, each accompanied by 95% uncertainty intervals. Red and green bars show the actual number of prevalence and YLDs cases, providing a clear visual comparison of age-specific burdens between genders. Abbreviations: TBI, Traumatic brain injury; ASPR, age-standardised prevalence rate; YLDs, years lived with disability.

In 2021, TBI prevalence increased across the 15–49-year age range and was consistently higher in males than females (Figure 2A–B). Among males, prevalent cases rose from 496,651 (95% UI 449,123–551,326) at ages 15–19 years to 2,462,402 (2,344,364–2,598,004) at 45–49 years, with ASPR increasing from 155.04 (140.21–172.11) to 1035.22 (985.60–1,092.23) per 100,000. Females showed lower levels, increasing from 255,942 (230,618–281,758) to 874,360 (831,300–922,097), with ASPR rising from 84.28 (75.95–92.79) to 371.05 (352.78–391.31) per 100,000. YLD rates followed the same direction, with higher levels in males than females.

For minor TBI, prevalence increased with age in both sexes (Figure 2C–D). In males, prevalent cases rose from 149,131 (125,778–175,669) at 15–19 years to 459,980 (424,476–495,165) at 45–49 years (ASPR 46.55 [39.27–54.84] to 193.38 [178.45–208.17] per 100,000); in females, cases increased from 85,932 (71,999–102,083) to 214,673 (196,922–233,917) (ASPR 28.30 [23.71–33.62] to 91.10 [83.57–99.27] per 100,000).

For moderate-to-severe TBI, males again showed higher prevalence across all ages (Figure 2E–F). Male cases increased from 347,520 (314,102–389,597) at 15–19 years to 2,002,422 (1,887,655–2,136,770) at 45–49 years (ASPR 108.49 [98.06–121.05] to 841.84 [793.59–898.32] per 100,000); female cases increased from 170,009 (152,015–190,541) to 659,687 (619,762–707,283) (ASPR 55.99 [50.06–62.75] to 279.95 [263.01–300.15] per 100,000).

### Contributions of risk factors to the TBI burden by SDI and age

**Figure 3.**
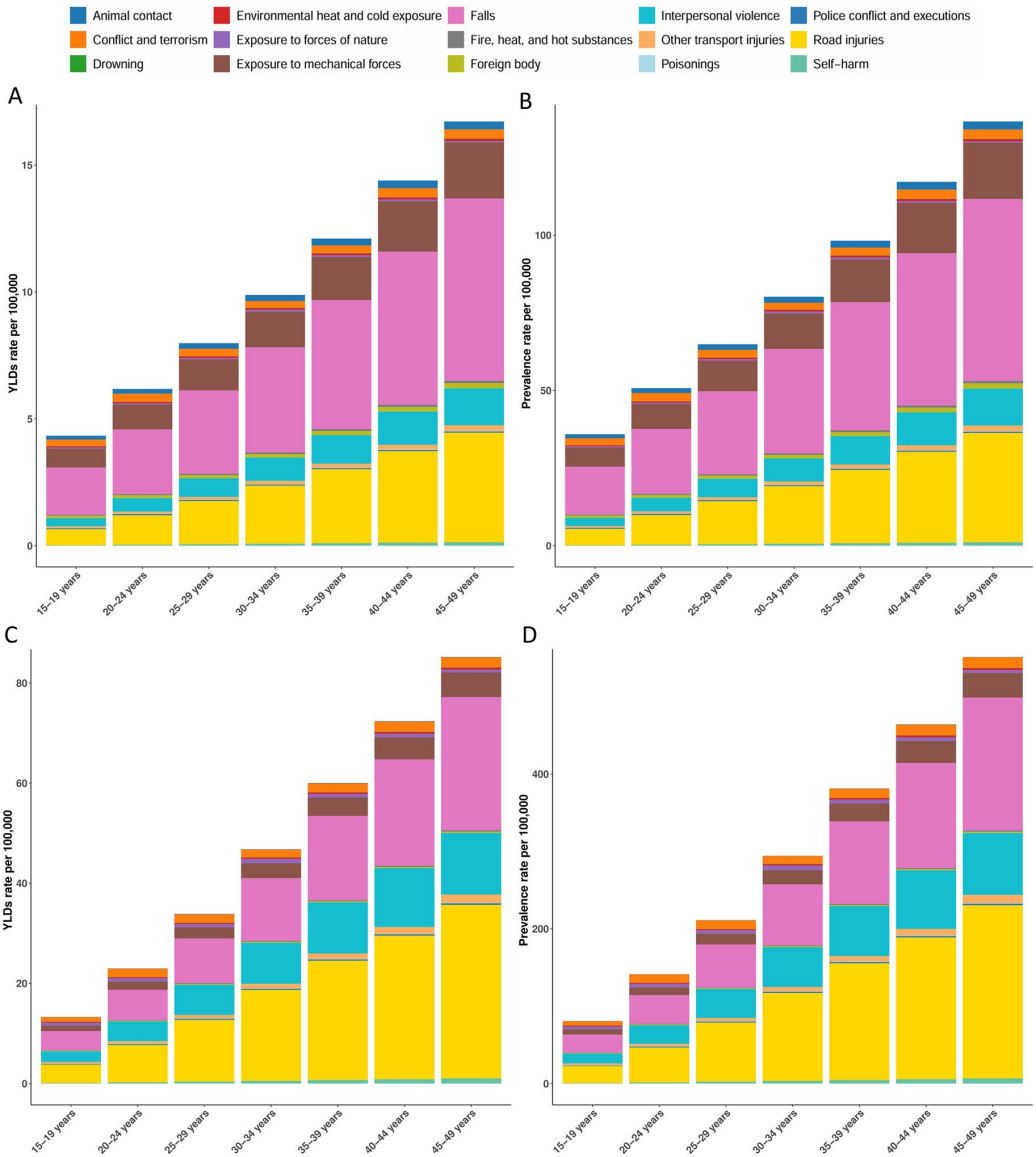
Contributions of risk factors to the global burden of TBI, by SDI and age group. Panels A and B show the contribution of individual risk factors to the age-standardised rate of YLDs and ASPR, respectively, across global and SDI quintile levels. Panels C and D present the same measures stratified by age group. Each colour represents a distinct risk factor contributing to the TBI burden. These stacked bar charts highlight the variation in risk factor contributions by development level and age. TBI: Traumatic brain injury; ASPR: age-standardised prevalence rate; YLDs: years lived with disability; SDI: sociodemographic index.

In 2021, risk-attributable TBI burden differed across SDI strata (Figure 3A–B). Globally, road injuries were the leading contributor to prevalence (128.97 per 100,000, 95% UI 120.85–140.88), followed by falls (116.38, 105.25–129.23) and interpersonal violence (54.58, 49.04–59.94). In middle SDI regions, road injuries exceeded falls (road injuries 148.63, 137.86–162.01 vs falls 120.14, 107.76–134.30). Conflict and terrorism contributed disproportionately in low SDI settings (45.75, 22.97–80.55) and remained evident in low-middle SDI regions (7.67, 3.82–13.67). For YLDs, in high SDI regions falls (21.68 per 100,000, 15.09–29.91) and road injuries (18.88, 12.86–25.83) were the dominant contributors, with analogous SDI gradients shown in Figure 3A–B.

Within the 15–49 year age range, causes varied by age (Figure 3C–D). Among 15–19-year-olds, falls were foremost (38.49 per 100,000, 31.81–46.26), followed by road injuries (27.36, 25.05–30.37) and interpersonal violence (15.36, 13.52–17.09). By ages 45–49 years, road injuries peaked at 258.96 per 100,000 (239.84–281.77) and falls reached 230.35 (206.87–256.07). Cause-attributable YLD patterns broadly mirrored prevalence, with falls and road injuries accounting for most disability burden across ages (Figure 3C–D).

### Trends and correlations of TBI burden by socio-demographic index levels

**Figure 4.**
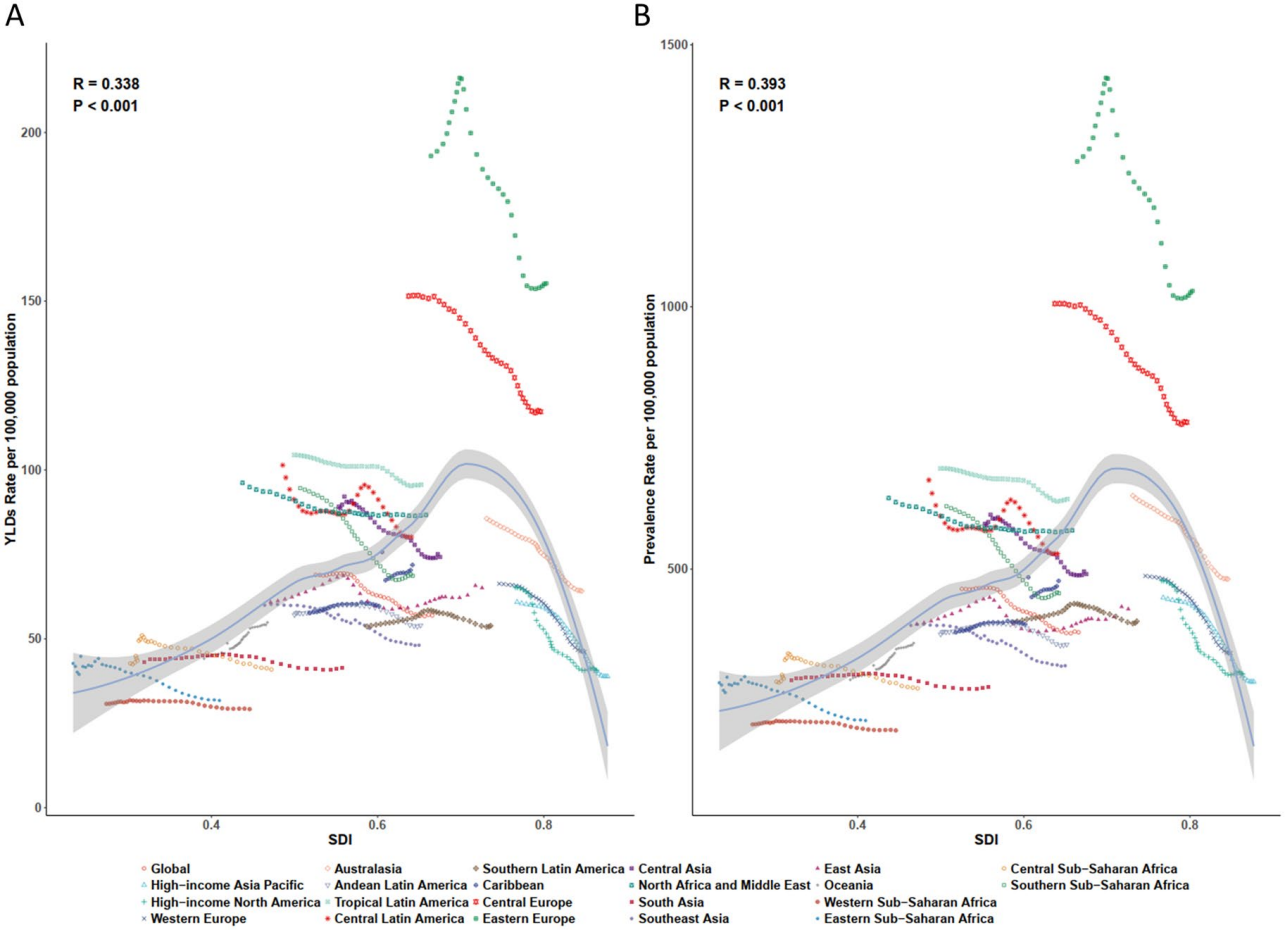
The age-standardised YLDs rate (A) and ASPR (B) of TBI by 21 GBD regions and Socio-demographic Index, 1990–2021. The solid line represents expected values calculated from SDI and aggregate disease rates. Thirty-two data points per region depict observed rates for each year; The shaded area shows the 95% uncertainty intervals of these expectations. Points above and below the line indicate higher and lower-than-expected disease burdens, respectively. TBI: Traumatic brain injury; ASPR: age-standardised prevalence rate; YLDs: years lived with disability; GBD: Global Burden of Diseases, Injuries, and Risk Factors Study; SDI: sociodemographic index.

At the regional level, the ASPR and age-standardised YLDs rate of TBI increased progressively with higher SDI levels (Figure 4). Several regions exhibited higher-than-expected TBI burdens relative to their SDI, notably Eastern Europe, Central Europe, and Central Asia. Notably, Eastern Europe recorded the highest ASPR, declining from 1277.37 (95% UI: 1208.87–1349.41) in 1990 to 1030.20 (95% UI: 973.26–1091.99) in 2021. Conversely, Southeast Asia and Western Sub-Saharan Africa demonstrated lower-than-expected TBI burdens considering their SDI levels. A strong positive correlation was observed between SDI and ASPR (*r* = 0.393, *p* < 0.001) and SDI and YLDs rate (*r* = 0.338, *p* < 0.001).

**Figure 5.**
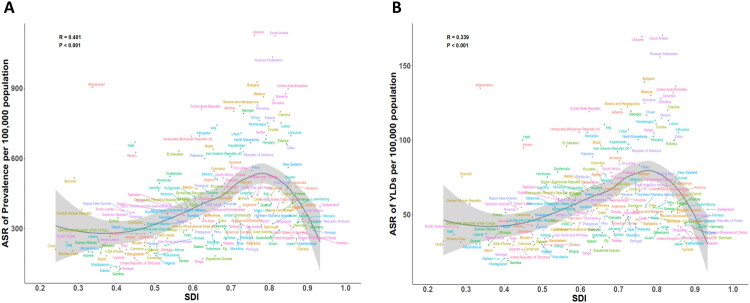
The ASPR (A) and age-standardised YLDs rate (B) of TBI by 204 countries and SDI, 2021. The solid line represents expected values calculated from SDI and aggregate disease rates. The shaded area shows the 95% uncertainty intervals of these expectations. Points above and below the line indicate higher and lower-than-expected disease burdens, respectively. TBI: Traumatic brain injury; ASPR: age-standardised prevalence rate; YLDs: years lived with disability; SDI: sociodemographic index.

At the national level, Ukraine, Saudi Arabia, and Bulgaria had significantly elevated ASPR and YLD rate beyond expectations based on SDI (Figure 5). Specifically, Ukraine presented the highest ASPR (1127.52 (95% UI: 1064.20–1200.26)) and age-standardised YLDs rate (169.70 (95% UI: 119.16–233.14)). Malawi, Madagascar, Senegal and Gambia consistently demonstrated lower-than-anticipated ASPR and YLD rates for their SDI classification. Correlation analysis revealed a positive correlation was observed between SDI and ASPR (*r* = 0.401, *p* < 0.001) and SDI and YLDs rate (*r* = 0.339, *p* < 0.001).

## Discussion

This study presents updated global, regional, and national estimates of TBI burden among individuals aged 15–49 years from 1990 to 2021. The total number of prevalent TBI cases increased substantially during this period, while both the ASPR and age-standardised YLDs rate showed modest declines. Regional variations were evident, with high-income regions experiencing reductions in TBI burden, whereas the Caribbean and Oceania showed increasing trends. National disparities were also observed, with countries such as Ukraine and Syria exhibiting higher-than-expected rates. Across all age groups, males consistently bore a higher burden than females. Prevalence and YLDs increased with age, peaking in the 45–49-year group. Road traffic injuries and falls were the predominant causes, while conflict and interpersonal violence contributed significantly in specific low-SDI settings.

The global trajectory of TBI burden over the past three decades reflects a complex interplay between demographic expansion, evolving patterns of exposure, and uneven progress in health system development [1,2,4]. Although the absolute number of TBI cases has increased markedly, both the ASPR and age-standardised YLDs rate have shown modest declines. This decoupling suggests that while preventive strategies and clinical management have improved at the population level, they remain insufficient or inconsistently implemented across regions [4]. Population growth—particularly in low- and middle-income countries, where young populations continue to expand—has substantially increased the number of individuals at risk [20]. At the same time, rapid urbanization and motorization have introduced new vectors for trauma, with road traffic incidents remaining the leading cause of TBI in many settings [21,22]. In the absence of comprehensive road safety legislation, enforcement systems, and injury surveillance, these demographic and structural transitions may have heightened vulnerability to TBI.

While this study focuses on adolescents and young adults (aged 15–49 years), we acknowledge the growing burden of TBI among older adults in high-income settings—an emerging public health priority driven by population ageing and increased fall risk [23]. However, the burden in younger populations warrants equal attention for three key reasons. First, the 15–49 year age group encompasses the most economically productive years; injuries sustained during this period result in the greatest loss of healthy life-years, with profound individual and societal consequences. Second, the aetiological profile of TBI in younger populations differs fundamentally from that in older adults—road traffic injuries and occupational hazards predominate—necessitating distinct prevention strategies [24]. Third, our findings on falls as a leading cause of TBI in younger adults highlight a risk factor that persists across the life course, providing a foundation for understanding fall-related TBI from young adulthood to old age. Future research should extend the GBD 2021 framework to conduct a similarly granular analysis of TBI burden in populations aged ≥ 70 years, incorporating age-specific considerations such as frailty, polypharmacy, and comorbidities. Such work, combined with our present analysis, would enable a comprehensive life-course perspective on TBI prevention and care [25].

In parallel, the observed declines in ASPR and YLD rates are likely driven by several converging improvements, particularly in high-income settings. These include the implementation of safety regulations—such as helmet and seatbelt laws, improved vehicle design, and enhanced emergency response systems—as well as greater public and clinical awareness of mild TBI [26,27]. Expanded access to diagnostic technologies and health information systems has also improved case detection [24,28]. These trends collectively highlight the urgent need to balance effective prevention with scalable rehabilitation strategies to address the growing burden of TBI-related disability worldwide.

Marked heterogeneity in the burden of TBI across regions and countries highlights the profound influence of context-specific dynamics—including conflict, socioeconomic instability, and health system resilience—on trauma epidemiology [1,29]. Divergent trends in ASPR and age-standardised YLDs rate across regions reflect substantial disparities in the capacity to prevent, manage, and recover from TBI.

In high-income regions such as North America, Western Europe, and the Asia-Pacific, sustained declines in ASPR and age-standardised YLD rates of TBI reflect long-term investment in trauma systems, safety regulations, and rehabilitation services [2,4]. These areas benefit from integrated care networks, strong policy enforcement, and wide access to specialist care [30]. Mature surveillance systems also support timely detection and data-driven policy adjustment. Conversely, the rising ASR in high-income Asia Pacific and North America, despite overall improvements, may reflect enhanced detection, ageing populations, or better case ascertainment. These trends suggest a dual burden of increasing recognition and persistent incidence [4].

Most low- and middle-SDI regions showed declining rates but rising absolute case numbers, driven by demographic growth. However, limited trauma care capacity, weak policy enforcement, and insufficient rehabilitation services may contribute to unmet needs. Strengthening prevention and post-injury care is crucial to reducing future burden [24,31].

At the national level, countries such as the Syrian Arab Republic and Burundi illustrate how armed conflict and political instability can significantly elevate TBI burden [4]. Injuries from explosive devices, blunt trauma, and interpersonal violence are common in such settings, where emergency response and post-injury care are often fragmented or unavailable [32]. By contrast, countries like Eritrea and Lebanon demonstrated sharp declines, though these may partially reflect underreporting due to surveillance limitations [33]. As such, reductions in burden must be interpreted cautiously, emphasizing the importance of triangulating estimates with independent metrics of health system performance.

The age- and sex-specific distribution of TBI burden reveals distinct demographic gradients in exposure risk and long-term consequences, shaped by biological, behavioral, and socio-cultural factors. Across the 15–49 year age range, both TBI prevalence and YLDs increase steadily with age, particularly among males, reflecting cumulative exposure to high-risk environments, occupational hazards, and age-related physiological decline [30,32]. Males consistently exhibited higher rates of both minor and moderate-to-severe TBI than females, a pattern widely reported in injury literature [34,35]. This disparity is likely multifactorial, involving greater participation in high-risk behaviors (e.g. reckless driving, alcohol use), increased representation in hazardous occupations, and higher rates of interpersonal violence and contact sports involvement. The sex gap emerges during adolescence and persists through mid-adulthood, underscoring the need for early, sex-specific prevention strategies.

The rising burden of minor TBI, especially in younger age groups, likely reflects both increased exposure and improved recognition. Heightened awareness of concussion and advances in diagnostic practices—particularly in sports and emergency care—have expanded case detection, though the long-term impact of mild TBI remains underappreciated. This growing cohort may face increased risk of post-concussive syndrome and neurocognitive impairment [36]. In contrast, the burden of moderate-to-severe TBI rises sharply in older adults within this age band, potentially driven by occupational risk, fall-related injuries, and comorbidities [37]. Among females, a lower but gradually increasing burden may reflect a different risk profile, shaped by gendered patterns of exposure (including unpaid household and caregiving activities that influence time spent in the home environment and related injury contexts), limited healthcare access, and underreporting influenced by cultural and gender norms [35].

The risk factor landscape for TBI highlights a complex intersection between socioeconomic development, injury exposure, and health system capacity. Globally, road traffic injuries and falls remain the leading contributors to TBI burden across all SDI strata. However, their relative impact—and the emergence of additional contextual risks—varies markedly by region, age group, and stage of development [24,38].

In high and high-middle SDI settings, the predominance of road injuries and falls reflects an ongoing epidemiological transition. Despite improvements in vehicle safety, urban infrastructure, and emergency response systems, these regions continue to report high rates of non-fatal trauma. Contributing factors include increasing urban density, ageing workforces, and widespread participation in recreational and high-risk activities [4]. While trauma systems may reduce mortality, improved survival rates lead to a growing population living with long-term disability, reflected in persistently high YLDs. By contrast, low and low-middle SDI regions face a broader and often more severe risk environment. In these settings, conflict and interpersonal violence are prominent causes of TBI, often compounded by underdeveloped emergency systems, delayed treatment, and poor access to rehabilitation [39,40]. These injuries are frequently underreported, yet contribute disproportionately to disability, functioning as a proxy indicator of systemic fragility and underinvestment in public health.

Notably, the relationship between SDI and TBI burden is non-linear. Countries such as Ukraine, Saudi Arabia, and Bulgaria exhibit burdens that exceed those predicted by their SDI level, possibly due to regional instability, occupational hazards, or inadequate safety regulation. Conversely, nations such as Madagascar, Malawi, and Senegal report lower-than-expected burdens, which may reflect underdiagnosis or data limitations rather than true reductions in risk.

Age-specific patterns further support the need for targeted prevention. Within the 15–49-year age range, falls were the leading cause of TBI among adolescents, plausibly reflecting sport and recreational injuries [41], school/workplace exposures (including falls from height) [42], and other environmental hazards, rather than the frailty-related mechanisms that typically characterise falls in older adults. From early adulthood onwards, road injuries became the dominant cause; however, fall-related TBI rates also increased with age and remained substantial in the older ages within this band [43], which reconciles differences in relative ranking with rising absolute rates across adulthood. These findings underscore the need for age-tailored prevention strategies that address both road safety and context-specific fall risks across adolescence and adulthood.

At the macro level, countries with higher SDI scores typically possess greater diagnostic capacity, comprehensive case detection, and robust reporting infrastructure—factors that increase the visibility of TBI, especially mild and moderate cases that might otherwise be overlooked. Accordingly, higher estimated prevalence and YLDs in high-SDI settings may partly reflect improved recognition and ascertainment, as well as better survival after severe injury, which can increase the number of individuals living with TBI-related disability, rather than intrinsically higher underlying risk [4]. Enhanced access to acute trauma care and rehabilitation services in these settings also contributes to improved survival following severe injuries, resulting in a growing population living with TBI-related disability [44]. In this context, a rising YLDs burden may paradoxically reflect success in trauma systems while simultaneously highlighting gaps in long-term rehabilitative care. Conversely, lower estimates in low-SDI settings may reflect underdiagnosis and incomplete capture of non-fatal TBI–especially mild TBI–due to constrained access to neuroimaging and weaker surveillance and registry systems.

Outliers within this trend provide important insights into the structural determinants of injury. Countries such as Ukraine and Bulgaria reported TBI burdens well above SDI-based expectations, potentially reflecting a combination of political instability, inadequate enforcement of road safety policies, underinvestment in injury prevention, and behavioral risk factors such as alcohol misuse and interpersonal violence [45]. In Ukraine, the escalation of armed conflict since 2022 has likely contributed to a higher burden of TBI, through increased blast-related injuries and disruptions to emergency and surgical care systems.

Conversely, several low- and middle-income countries—including Malawi, Senegal, and Madagascar—exhibited lower-than-expected TBI burdens. Although this may reflect genuine differences in exposure to mechanized transport or industrial injury, it is more plausibly attributed to underdiagnosis and data limitations [46]. Weak surveillance systems, particularly for non-fatal and mild TBI, may distort estimates, leading to under-recognition of true disease burden and insufficient policy prioritization [47]. These findings emphasize the need to interpret SDI correlations with caution, recognizing the influence of health system maturity, governance, and structural inequities. Strengthening surveillance infrastructure—especially in low-SDI countries—is essential for accurate burden estimation and to guide equitable investment in prevention, acute care, and rehabilitation for TBI.

This study provides the most extensive and current assessment of the TBI burden among adolescents and young adults to date, covering 204 countries and territories from 1990 to 2021. The use of the GBD 2021 framework, Bayesian meta-regression modelling, and standardised metrics enables robust comparisons over time and across regions. Stratified analyses by age, sex, and SDI allow for nuanced interpretation and support precision health policy development. Nonetheless, certain limitations should be acknowledged. First, the burden of TBI is likely underestimated in LMICs due to underreporting, limited diagnostic infrastructure, and weak surveillance systems; such differential ascertainment across settings may bias comparisons by SDI/income and should be considered when interpreting observed gradients. Second, reliance on modelled data introduces uncertainty, especially in data-scarce regions. Third, the minor vs moderate-to-severe stratification, used for GBD comparability, is necessarily coarse and may not reflect individual trajectories, potentially introducing misclassification and attenuating severity-specific contrasts. Finally, as an ecological study, our analysis cannot establish causality between TBI burden and associated risk factors or sociodemographic variables.

## Conclusion

This study highlights a rising global burden of traumatic brain injury among adolescents and young adults, despite modest declines in age-standardised rates. Marked disparities across regions, sexes, and socio-demographic levels underscore the need for tailored prevention, improved surveillance, and expanded access to trauma care and rehabilitation. Addressing both the incidence and long-term consequences of TBI will require coordinated, context-specific strategies to reduce inequities and support sustainable health system strengthening.

## Supplementary Material

STROBE.pdf

## Data Availability

The data that support the findings of this study are available from the corresponding author, Kai Zhang, upon reasonable request.
